# Characterizing ER Retention Defects of PDZ Binding Deficient Cx36 Mutants Using Confocal Microscopy

**DOI:** 10.21769/BioProtoc.5034

**Published:** 2024-07-20

**Authors:** Stephan Tetenborg, Elizabeth Martinez-Soler, John O`Brien

**Affiliations:** College of Optometry, University of Houston, Houston, TX, USA

**Keywords:** Connexin 36, ER retention, trafficking, HEK293T cell, PDZ

## Abstract

Overexpression of proteins in transiently transfected cells is a simple way to study basic transport mechanisms and the underlying protein–protein interactions. While expression systems have obvious drawbacks compared to in vivo experiments, they allow a quick assessment of more conserved functions, for instance, ER export or sorting of proteins in the Golgi. In a previous study, our group described the formation of ER-derived removal vesicles for the gap junction protein Cx36 in transfected HEK293T cells. These removal vesicles, termed “whorls” because of their concentric structure, were formed by Cx36 channels that failed to escape the ER. In this article, we describe an imaging protocol that can be used to determine these ER retention defects for Cx36 expressed in cultured cells. The protocol we provide here employs regular confocal microscopy, which allows for sufficient resolution to reveal the characteristic shape of ER whorls.

## Background

**Figure 1.**
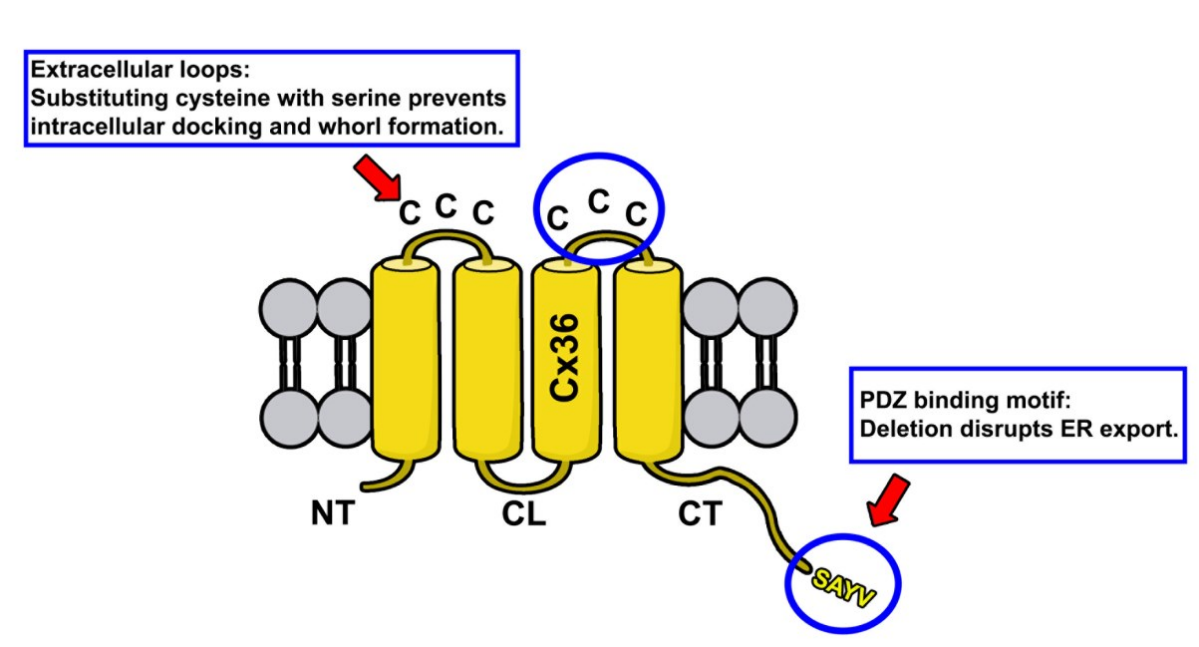
Membrane topology of Cx36. Protein structure of Cx36 is illustrated. The two extracellular loops of Cx36 (indicated by upper circle and red arrow) contain three cysteines. The C-terminal tail contains the PDZ binding motif consisting of the following amino acid sequence: SAYV.

Gap junctions are clusters of intercellular channels that directly connect the cytoplasm of adjacent cells, providing a conduit for the exchange of metabolites and ions. In chordates, gap junctions are formed by connexins, a diverse family of membrane proteins that have the ability to oligomerize into dodecameric channels. Mutations in connexin genes are the cause of a variety of inherited diseases. In many cases, these pathologies have been linked to trafficking defects that compromise the ability of the channel to form functional gap junctions [1,2]. Among the 21 connexins that have been identified in humans, only a few variants have been studied in terms of trafficking through intracellular compartments [2–4]. To study basic transport mechanisms of gap junction proteins, researchers take advantage of expression systems, such as HeLa or HEK293T cells, and transfect these cells with recombinant expression vectors. In this article, we describe a detailed protocol that can be used to determine endoplasmic reticulum (ER) retention defects for connexin 36 (Cx36). In a previous study, we described a transport defect that prevented the functional ER export of Cx36, causing the connexin to accumulate in the ER [5]. This retention mechanism promoted the formation of gap junction-like aggregates that reshaped the ER into concentric multi-membrane vesicles (Figures 1B and D), which we termed connexin whorls. These structures were characterized by several distinct features: 1) Each sheet within the whorl exhibited ultrastructural features that were indistinguishable from an actual gap junction; 2) whorls are hollow inside and their diameter varied, ranging from 0.3 to 3 µm; 3) whorls colocalized with ER-phagy receptors Tex264 (Testis-expressed protein 264) (Figure 1C) and p62; 4) whorl formation requires docking interactions of extracellular loops in Cx36 facing the ER lumen. Substituting the extracellular loop cysteines (C55 or C62) via site-directed mutagenesis prevents whorl formation. Similar whorl-like structures were reported for the lens connexin Cx50 in a previous study by Lichtenstein et al., [6], which suggests that ER-derived whorls are formed by many connexins that oligomerize in ER. Therefore, the protocol we describe here is not only applicable for Cx36 but might be used for other connexin isoforms. However, two important aspects have to be considered: 1) Some connexins, for instance Cx43, oligomerize in the Golgi [7], which would make the docking of Cx43 containing connexons in the ER impossible; and 2) whorls have to be tested for the presence of ER proteins, for instance Tex264, to determine the compartment they originated in (Figure 1C).

**Rationale for experimental design**

The rationale for the design of connexin mutants we have described in Tetenborg et al., (2023) was based on the observation that truncation of the C-terminal tip in Cx36 prevents functional ER export leading to the intracellular formation of connexin whorls. To test if these multimembrane vesicles are formed by a gap junction-like docking mechanism of opposing hemichannels in the ER, we substituted cysteines in the extracellular loop of Cx36 with serines via site-directed mutagenesis. These mutations were previously shown to block gap junction formation [8] and served as a control experiment in our study to confirm that ER retention causes opposing Cx36 channels to dock intralumenally via the extracellular loops.

## Materials and reagents

**Biological materials**

1. Anti Cx35/Cx36 antibody (depends on the connexin that is studied); our study focused on Cx36 (Sigma-Aldrich, catalog number: MAB3045)
2. Anti Testis expressed protein 264 (Tex264) (Novus Biologicals, catalog number: NBP1-89886)
3. Anti Golgin97 (Thermo Fisher Scientific, catalog number: PA5-30048)
4. Human embryonic kidney 293T cells T/17 (ATCC, catalog number: CRL-11268)
5. Connexin 36 expression vector (or any other connexin), map shown in Figure 1
6. Normal donkey serum [9] (Jackson Immunoresearch, catalog number: 017-000-121)
7. Donkey anti-mouse Cy3 (Jackson Immunoresearch, catalog number:715-165-160)
8. Donkey anti-rabbit Alexa Fluor 488 (Jackson Immunoresearch, catalog number:715-545-152)

**Reagents**

1. Dulbecco’s modified Eagle’s serum (DMEM) (Thermofisher, catalog number: 12800017)
2. Fetal bovine serum (FBS) (Thermofisher, catalog number: A5209401)
3. 2.5% Trypsin 10× (Thermofisher, catalog number: 15090-046)
4. Geneporter 2 (Amsbio, catalog number: T20200)
5. Paraformaldehyde (PFA) 20% (Electron Microscopy Sciences, catalog number: 15712)
6. Poly-l-lysine (PLL) 0.01% (Millipore, catalog number: A-005-C)
7. Triton-X 100 (Fisher Scientific, catalog number: BP151-100)
8. 50 mL Falcon tube (Falcon, catalog number: 352070)
9. Phosphate buffered saline (PBS) (Sigma-Aldrich, catalog number: P3813)
10. Vectashield PLUS mounting media, DAPI (Vector Laboratories, catalog number: H-2000)
11. Tissue Path Superfrost^TM^ Plus gold (Thermofisher, catalog number: 1518846)
12. Normal donkey serum (NDS) (Jackson Immunoresearch, catalog number: 017-000-121)

**Laboratory supplies**

1. 75 cm^2^ cell culture flask (Corning, catalog number: 430725U)
2. 35 mm × 10 mm style cell culture dish (Corning, catalog number: 430165)
3. Coverslips (Fisher Scientific, catalog number: 12541000)
4. 24-well plate (Corning Incorporated, catalog number: 3526)
5. Axygen^TM^ Maxyclear Snaplock microtubes, 1.5 mL (Fisher Scientific, catalog number: 14-222-158)
6. Nail polish, L.A colors, rapid dry (Electron Microscopy Sciences, catalog number: 72180)

## Software and datasets

1. Fiji, open source [10]

## Equipment

1. Biological safety cabinet, 1300 Series A2 (Thermofisher, catalog number: 72180)
2. Incubator (Eppendorf, model: Galaxy 170 S)
3. Eppendorf research plus pipette (Eppendorf, catalog number: 3123000012)
4. Confocal laser scanning microscope (Zeiss, model: LSM800)

## Procedure

- **Day 1: Seed HEK293T cells**
  1. Culture HEK293T cells in DMEM supplemented with 10% FBS and 1% penicillin and 1% streptomycin at 37 °C in a humidified atmosphere with 5% CO_2_. Grow cells to confluence prior to the experiments.
  2. Transfer 2–3 coverslips to 35 mm dishes and incubate in 0.01% PLL for 30 min at RT.
  3. Remove PLL and briefly wash the coverslips in sterile water.
  4. Prepare 10 mL of a 0.25% Trypsin solution in serum-free DMEM (9 mL of DMEM + 1 mL of 2.5% trypsin).
  5. Remove the media from the cell culture flask and briefly wash the cells with serum-free DMEM.
  6. Apply 10 mL of the trypsin solution and incubate the cells at 37 °C for 10 min in the incubator.
  7. Transfer the dissociated cells to a 50 mL Falcon tube and centrifuge at 1,500× *g* for 10 min at room temperature.
  8. Remove the supernatant and resuspend the cells in 5–10 mL of serum-free DMEM.
  9. Determine the number of cells per milliliter using a hemocytometer.
  10. Apply 450,000 cells to coated coverslips. Apply 2 mL of DMEM containing 10% FBS and incubate overnight.
- **Transfect HEK293T with the Cx36 expression vector**
  1. Twenty-four hours after seeding, remove the media and briefly wash the cells with serum-free DMEM. Apply 900 µL of serum-free DMEM and place the cells back in the incubator.
  2. Prepare the transfection reaction consisting of **mix A** and **mix B.**
**Mix A:** Combine 5 μL of Geneporter 2 with 43 μL of serum-free DMEM.
**Mix B:** Combine 1 μg of the Cx36 expression vector (Figure 2) with 50 μL of DNA diluent (Diluent A or B).
  3. Combine Mix A and Mix B and incubate for 15 min at RT.
  4. Apply the transfection mix to HEK293T cells and incubate for 2 h.
  5. Two hours after step B4, apply 1 mL of 20% FBS DMEM and incubate the cells overnight.
- **Prepare cells for confocal microscopy**
  1. Nineteen to twenty-four hours after transfection, transfer the coverslips to a 24-well plate and briefly wash in PBS.
  2. Fix the cells in 2% PFA in PBS for 15 min at RT.
  3. Wash the cells 3 × 10 min with PBS at RT.
  4. Dilute the Cx36 antibody 1:500 in PBS containing 0.5% Triton-X 100 and 10% NDS.
    Additionally, the Cx36 antibody can be combined with a Tex264 (1:200) and a Golgin97 antibody (1:100) for double-labeling experiments.
  5. Apply the antibody solution to the well containing the coverslip and incubate overnight.
  6. The next day, wash the coverslips 3 × 10 min with PBS.
  7. Dilute the secondary Cy3-conjugated antibody in PBS containing 0.5% Triton-X and 10% NDS. When Tex264 and Golgin97 are double labeled with Cx36, a secondary conjugated with Alexa488 is needed.
  8. Incubate for 1 h under light-protected conditions at RT.
  9. Wash the coverslips 3 × 10 min with PBS at RT.
  10. Apply 5 μL of mounting media containing DAPI to an object slide. Mount the coverslips with the cell side facing the mounting media.
  11. Seal the coverslips with nail polish.
    **Figure 2.**
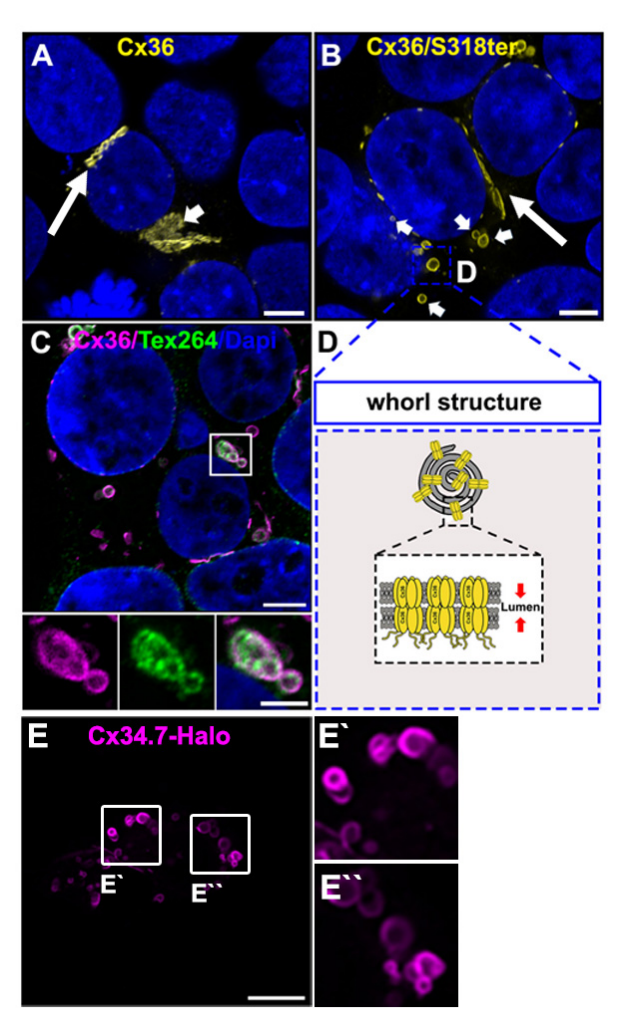
Connexin whorls. A. Transiently transfected HEK293T cells expressing Cx36. Cx36 is concentrated at gap junctions, indicated by the long arrow. Cx36 is also visible in perinuclear structures resembling the Golgi apparatus. Indicated by the short arrow. Scale: 5 μm. B. Transfected HEK293T cell expressing the trafficking deficient Cx36/S318ter mutant. Cx36 is retained in the ER and accumulates in ER whorls, which are labeled with short arrows. Gap junctions are still formed by the Cx36/S318ter mutant. Labeled with the long arrow. Scale: 5 μm. C. Example of Cx36 whorls colocalizing with the ER phagy receptor Tex264. Scale: 5 μm. Magnified inset: 2.5 μm. D. Cartoon illustrating the composition of ER whorls. E. Whorls are seen for different connexins. Confocal scan of Halo-tagged zebrafish Cx34.7, an orthologue of Cx36. Scale: 10 μm.
- **Confocal microscopy and image analysis**
  1. Image Cx36-transfected cells containing whorls using the Zeiss LSM800 confocal laser scanning microscope (or any other model with sufficient resolution) and 63× oil objective. Choose the correct laser settings to detect Cy3 (labeling Cx36) and DAPI. Use 2–3× zoom and a pixel size of ~50 nm × 50 nm. Scan a sufficient number of cells for each experimental condition.
  2. Images can be acquired as stacks of 1 with a spacing of 0.2 µm.
  3. Open your confocal scans in ImageJ (Fiji) and select individual Cx36 whorls using the rectangular selection tool (Figure 4).
    **Figure 3.**
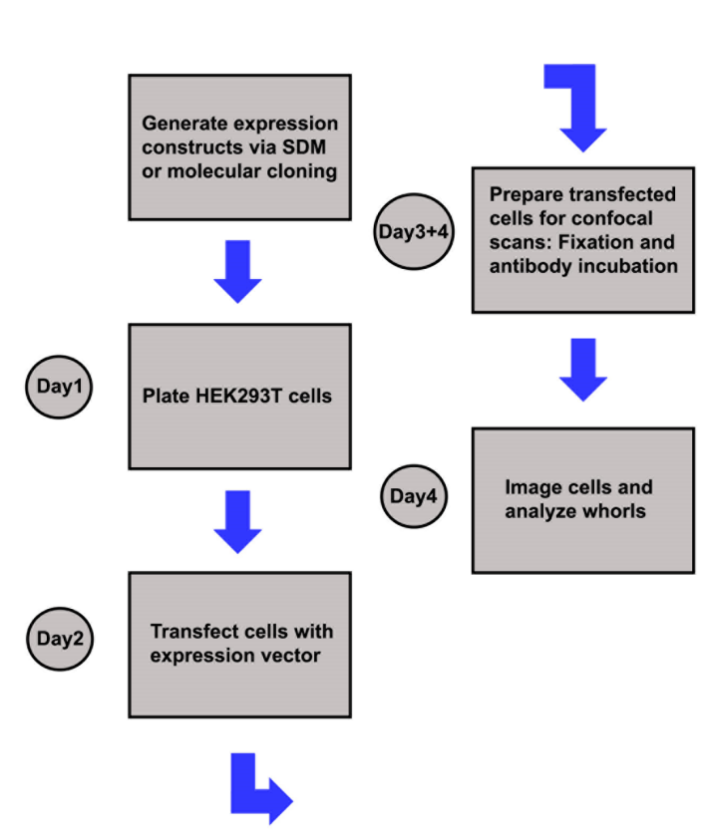
Sketch illustrating the experimental workflow
  4. Duplicate the region of interest (Click shift + D).
  5. Draw a straight line using the line tool and use the measure function to determine the diameter of the whorl.
- **Determine the number of whorls per cell**
  1. Based on the previous measurements, determine a size threshold to exclude smaller vesicles from quantification. As described in Tetenborg et al., [5], vesicles with a diameter smaller than 0.3 μm were not considered whorls.
  2. Open the cell counter plugin (Figure 5) and click *initialize*.
  3. Select a type of counter and count the whorls in your stack via left clicks.
    **Figure 4.**
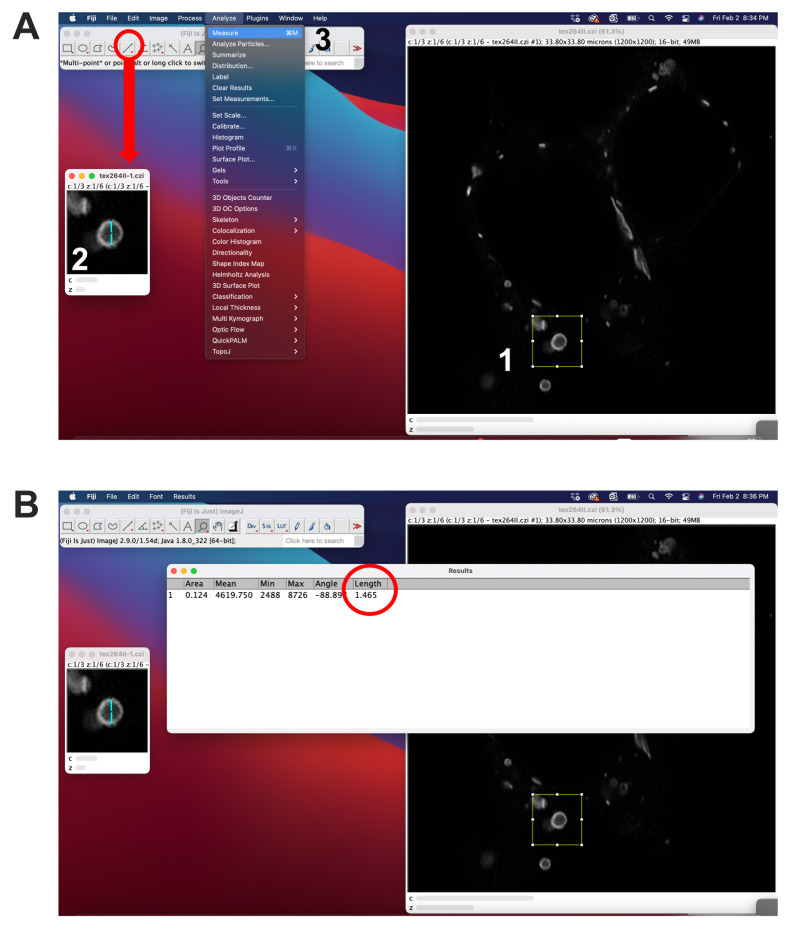
Measurement of whorl diameter using ImageJ. A. 1) A region of interest (ROI) surrounding the whorl can be selected using the rectangular selection tool. The ROI can be duplicated (shift + D). 2) To measure the diameter of a selected whorl, draw a line using the line selection tool and 3) use the measure function (Ctrl + M). B. The length will be displayed in the last column.
    **Figure 5.**
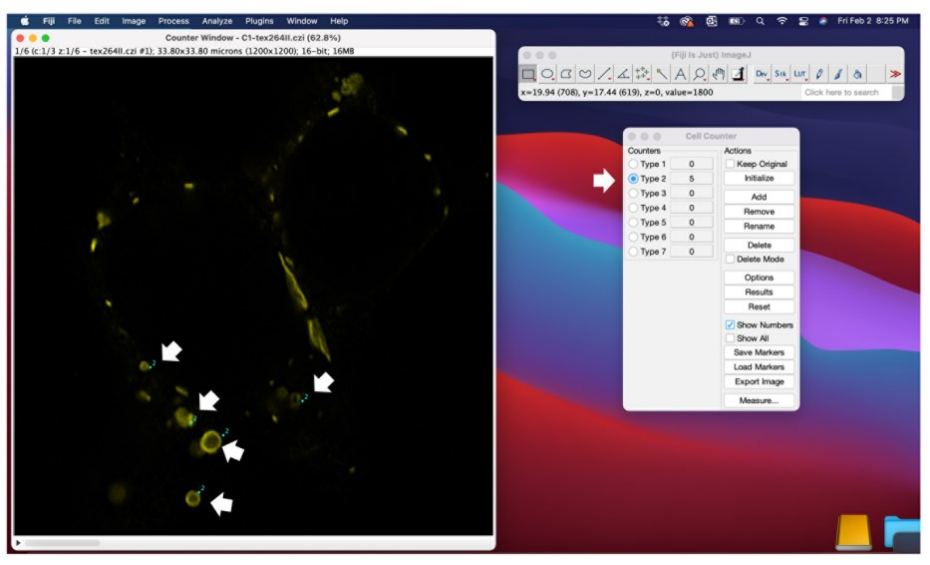
Quantification of ER whorls using the cell counter function in ImageJ. Confocal scans can be opened in ImageJ. Before the quantification, several scans of the experiment should be inspected to determine a size threshold for whorls (see Figure 3). Every vesicle with a diameter under the size threshold is excluded from quantification. The cell counter plugin has to be initialized in order to select vesicles that are counted. Each vesicle that is considered a whorl based on shape and diameter can be selected by a mouse click.

## Validation of protocol

This protocol or parts of it has been used and validated in the following research article(s):

Tetenborg, S. et al. (2023). Intralumenal docking of connexin 36 channels in the ER isolates mistrafficked protein. J Biol Chem. (Figure 1, panel C-E, Quantification of 11-30 vesicles, from 9-11 cell clusters)
